# Characterizing experiences of non-medical switching to trastuzumab biosimilars using data from internet-based surveys with US-based oncologists and breast cancer patients

**DOI:** 10.1007/s10549-022-06615-2

**Published:** 2022-05-14

**Authors:** Elizabeth Lerner Papautsky, Martha Carlson, Sheila M. Johnson, Hannah Montague, Deanna J. Attai, Maryam B. Lustberg

**Affiliations:** 1 Department of Biomedical & Health Information Sciences, University of Illinois at Chicago, 1919 W. Taylor St., Chicago, IL 60612 USA; 2 Chicago, IL USA; 3 Swansea, IL USA; 4 Department of Biomedical & Health Information Sciences, University of Illinois at Chicago, Chicago, IL USA; 5 Department of Surgery, University of California Los Angeles, Los Angeles, CA USA; 6 Yale Comprehensive Cancer Center, Yale School of Medicine, New Haven, CT USA

**Keywords:** Breast cancer, Biosimilars, Trastuzumab, Patient-centered care, Patient-provider communication, Communication

## Abstract

**Purpose:**

To characterize current experiences with communication and decision-making practices when non-medical switching to a biosimilar trastuzumab is proposed or required by cancer center or insurer.

**Methods:**

We developed and launched 60- and 51-item internet surveys to elicit US breast cancer patient and medical oncologist lived experiences with trastuzumab biosimilars and patient information needs and seeking practices. We recruited participants using social media and administered via REDCap in 2020–2021.

**Results:**

143 breast cancer patients and 33 medical oncologists completed the surveys. 63.9% patients reported having switched to a trastuzumab biosimilar and 40.8% reported receiving no prior notification about switching. 44% of patients reported learning about biosimilars primarily through self-directed learning and 41% wanting more time to discuss with oncologist. None of the oncologists reported that the decision to switch a patient to a biosimilar was initiated by them, but rather more frequently by the insurer (45.2%). About 54.8% reported not receiving any pharmaceutical manufacturer material related to the selected biosimilar. Patients and oncologists diverged in their responses to items regarding patient opportunities to ask questions, adequacy of resources, effectiveness of treatment, patient worry, and magnitude of change.

**Conclusion:**

There is a need for tailored and effective patient and oncologist information and education on trastuzumab biosimilars, along with improved healthcare communication regarding switching. The discrepancy between patient-reported experiences and oncologist perceptions of the patient experience, suggests a lack of adequate information that may be a challenge not only to the uptake of trastuzumab biosimilars, but to the patient-oncologist relationship.

**Supplementary Information:**

The online version contains supplementary material available at 10.1007/s10549-022-06615-2.

Trastuzumab, approved in 1998, has been revolutionary in improving survival for early stage and metastatic HER2-positive breast cancer. Trastuzumab is a monoclonal antibody—a biologic agent cultured from cells grown in manufacturing facilities [1]. However, its high cost increases cancer care expenses, presenting challenges to healthcare systems and patients [2]. Biosimilars, which are biological products that are highly similar to the reference product in terms of purity, molecular structure, and bioactivity [3], are increasingly used in many areas of medicine, including oncology. The approval of trastuzumab biosimilars by the Food and Drug Administration (FDA), 5 as of 2019 (Kanjinti, Ontruzant, Ogivri, Trazimera, and Herzuma), is promising in terms of economic benefits of cost savings (production costs < 20–30% than reference) to healthcare systems and health benefits of potential access to life-saving treatment for more patients [2]. The US market for biosimilars is expanding, with large hospital systems and insurers choosing biosimilar products. In a 2018 statement by the American Society of Clinical Oncology (ASCO), authors highlight their commitment to education and guidance on the use of biosimilars that are equally efficacious to the reference medication to the oncology community [4], reaffirmed in 2022 [5]. Current ASCO guidelines reflect the use of any available formulations of trastuzumab, including biosimilars [6]. A set of 2021 guidelines by National Comprehensive Cancer Network (NCCN) addresses challenges of payer authorizations, stocking, and procurement errors [7].

Non-medical switching from the original FDA-approved medication to a biosimilar may be dictated by insurers or healthcare systems due to financial reasons. Biosimilars have become more commonly used for multiple disease types. Literature suggests that with increasing availability of biosimilars, a variety of switching scenarios have become common across disease types. With guidelines often being vague, the practice of switching is largely unregulated [8]. There is a critical need to account for the patient experience in switching to biosimilars. In a sample of 1696 surveyed patients diagnosed with Crohn’s disease, psoriasis, and other illnesses, 85% reported concerns regarding biosimilar effectiveness and 83% about side-effects [9]. In addition, patients have reported lack of awareness, education, and comprehension issues [10]. Medical oncologists have reported uncertainty about switching, lack of confidence in biosimilars, and existing financial incentives favoring the original biologic. In a 2019 survey, 34% of medical oncologists reported safety concerns [11]. Two 2021 international surveys (Brazil and Turkey) with medical oncologists documented concerns associated with switching [12], with the Turkish survey reporting that over half of the participants object to a switch from a reference product [13]. A 2020 survey with US oncologists found that knowledge about biosimilars was low and that community and private practice oncologists were more worried about safety and efficacy than ones practicing in academic medical centers [14].

Despite high uptake of biosimilars, knowledge about basic features of biosimilars was low, and oncologists in community and private practice settings were more often concerned about safety and efficacy than those in academic practices.

We wanted to characterize the experiences of patients and oncologists when switching to a biosimilar trastuzumab from the reference medication is proposed or required by cancer center or insurer. We were specifically interested in whether patients reported higher levels of concern related to the potential/actual switching than oncologists perceived them to have. Additionally, we anticipated that patients may engage in self-directed information seeking in the absence (or limited) information from their cancer care team. Thus, we developed and launched internet surveys to elicit patient and medical oncologist experiences in this emerging and common area of oncology practice.

## Materials and methods

### Instruments

We developed two distinct surveys to characterize experiences with trastuzumab biosimilars. One, containing 60 items, was designed for US patients with a diagnosis of HER2-positive breast cancer. The other, containing 51 items, was designed for US medical oncologists. Both surveys were drafted by a patient advocate (MC) and oncologist (MBL) and refined with support from a social scientist (ELP). Patient and oncologist surveys are included in Supplementary Materials. We piloted and further revised the surveys based on feedback from 4 patient advocates and 2 oncologists.

Survey recruitment posts were shared by all, but one of the authors using social media (including Twitter and Facebook) targeting their social networks and general oncology and breast cancer-specific patient communities. Medical oncologists known to the study team were also directly emailed to solicit participation. Additionally, approximately 3000 medical oncologists across the US (list purchased through Medical Marketing Services, Inc) were recruited using an e-blast. We employed multiple strategies to overcome recruitment challenges including posting advertisements at continuous intervals, utilizing multiple wording/phrasing options, and developing and utilizing an infographic and a recruitment video.

The survey was administered electronically using Research Electronic Data Capture (REDCap) hosted at the University of Illinois at Chicago [15]. Information about the survey was included on the landing page. Participants indicated consent by proceeding to the survey. Surveys comprised demographics and items specifically developed for this study that characterized lived experiences with trastuzumab biosimilars. Patients were also asked about their information needs and seeking practices. Participants were able to skip items. Estimated mean completion time for each survey was approximately 10 min. Participants were not offered compensation and no personally identifying information was collected. The patient survey was open from 8/26/2020–4/26/2021, and the medical oncologist survey was open from 8/26/2020–10/12/2021.

### Statistical methods

All analyses used SPSS version 28 statistical software [16] and Microsoft Excel 2019 [17]. We calculated descriptive statistics of counts, proportions, means, and standard deviations. Given that participants were able to skip items, we handled missing values on the basis of individual item to avoid excluding participants. In addition, we also identified illustrative quotes elicited as part of open-ended items. All study procedures were reviewed and approved by the University of Illinois at Chicago’s Institutional Review Board (Protocol #2020-0859; Exemption Granted: 7/29/20).

## Results

### Demographics

**Fig. 1:**
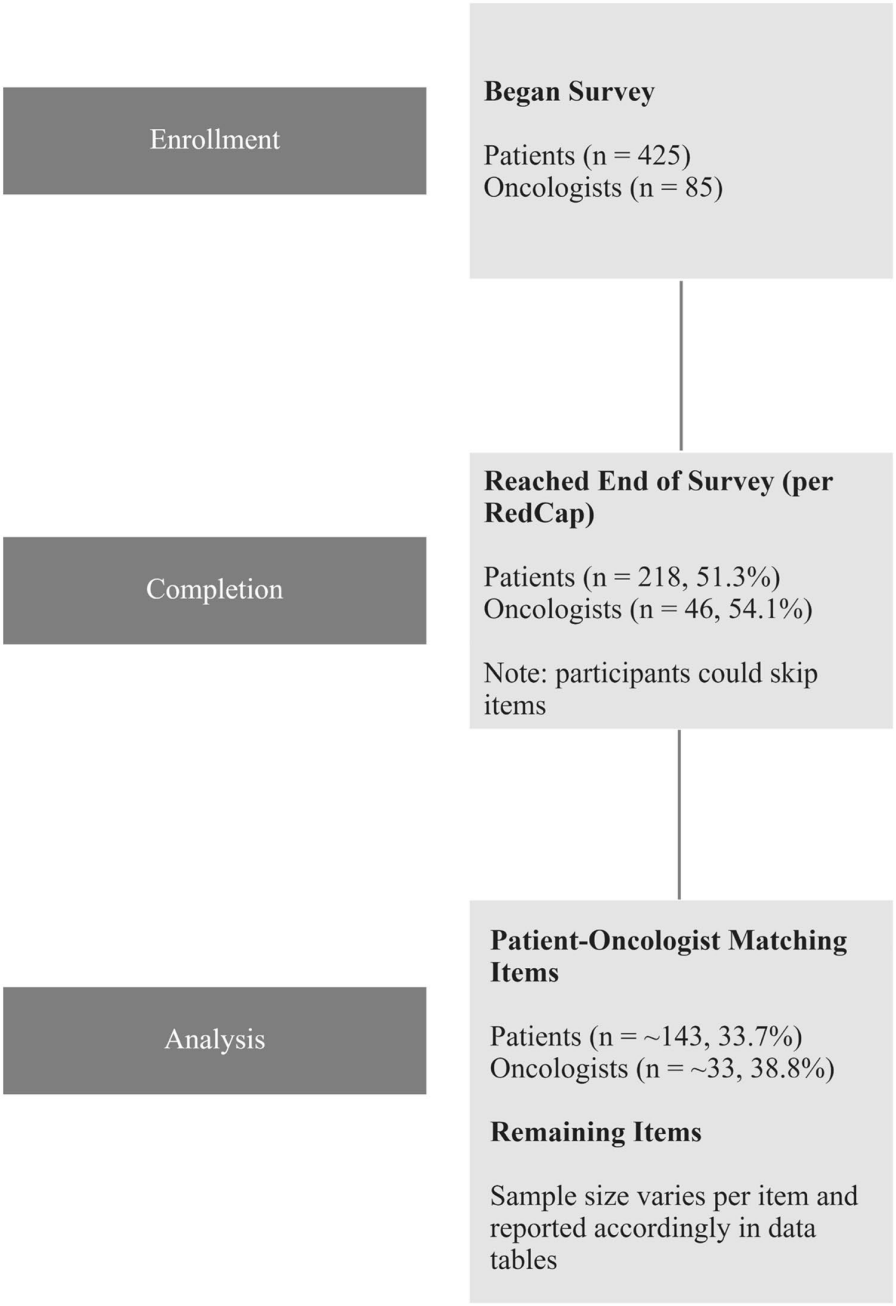
Flow diagram representing number and proportion of participants who began and completed their surveys, as well as sample sizes analyzed

See Fig. 1 for completion rates of the surveys. Sample sizes varies per item for patients and oncologists. Slightly less than half of the patients and oncologists began but did not complete the survey. We reported the sample sizes accordingly in the results tables. Average age was 49.59 (range = 30 - 77) years for patients and 46.16 (range = 32 - 81) years for oncologists. The median number of years that oncologists have been providing treatment was 10.00 (range = 0 - 35). Respondents were (patients; oncologists, respectively) White (91.4%; 38.8%), Black (4.8%; 1.2%); and not Hispanic (94.6%; 82.0%). Over half of the patients (56%) reported having a bachelor’s degree. Top 3 US states represented in the sample for patient residency are California (12.7%), Florida (8.3%), and Michigan (6.6%); for oncologist residency are Ohio (30.6%), Florida (12.2%), and New York (10.2%). Complete demographics for patients and oncologists are shown in Table 1.

**Table 1:** Demographics for patients and oncologists

	Patients		Oncologists	
Age	M = 49.59 (SD = 10.98)		M = 46.16 (SD = 11.04)	
	Percent (%)	frequency/N	Percent (%)	Frequency/N
Female	99.3	186/186	68.0	
White	91.4	171/187	38.8	33/85
Black	4.8	9/187	1.2	1/85
American Indian or Alaskan Native	1.1	2/187	1.2	1/85
Asian or Pacific Islander	2.7	5/187	11.8	10/85
Hispanic	3.8	7/185	10.0	5/50
Bachelor's Degree	> 56	65/186		

In Table 2, we report descriptive statistics associated with patient insurance and cancer-specific factors and patient and oncologist treatment/practice setting. The majority (58.1%) of patients reported being fully covered by private insurance, receiving care in a suburban area (54.4%), and being treated at a community oncology setting (44.1%). Patients reported a median of 1.62 (range = 0 - 41) years since first cancer diagnosis and 1.5 (range = 0 - 17) years since metastatic cancer diagnosis. Over half (56.3%) reported having a diagnosis of metastatic breast cancer; 84.8% having received treatment with trastuzumab within the past year. Oncologists reported practicing in an urban center (68.0%) at an academic medical center or affiliate (35.3%).

**Table 2:** Patient and oncologist background

	Patients		Oncologists	
	Percent (%)	frequency/n	Percent (%)	frequency/n
*State of residence (top 3)*				
Top 1	CA: 12.7	23/181	OH: 30.6	15/49
Top 2	FL: 8.3	15/181	FL: 12.2	6/49
Top 3	MI: 6.6	12/181	NY: 10.2	5/49
*Insurance*				
Fully covered by private insurance	58.1	108/186		
Partially covered by private insurance	15.1	28/186		
Medicare	18.3	34/186		
Medicaid	4.3	8/186		
Self-Pay	0.5	1/186		
*Area receiving/providing care*				
Urban	36.3	66/182	68.0	34/50
Suburban	54.4	99/182	28.0	14/50
Rural	9.3	17/182	4.0	2/50
*Care/practice setting*				
University-affiliated cancer center or its satellite location	22.0	41/186	36.5	31/85
Non-university affiliated cancer center (e.g. Cancer Centers of America, etc.)	15.6	29/186	3.5	3/85
Oncologist's office or community oncology setting	44.1	82/186	17.6	15/85
Community hospital	14.0	26/186	1.2	1/85
Veterans Affairs (VA) hospital	0.0	0/186	0.0	0/85

### Patient and oncologist experiences

Approximately 55.2% of patients who responded to this item reported being presented with an option to switch from reference trastuzumab to biosimilar. More than half of the patient respondents (63.9%) reported having switched to a trastuzumab biosimilar, most commonly Kanjinti (69.8%) and 8.6% declined the switch. About 40.8% of patients reported receiving no prior notification about switching, while 26.4% reported the treating physician or oncologist first discussed biosimilars with them, while others reported that information came from an advanced practice provider (5.7%), chemotherapy nurse (15.5%), pharmacist (2.9%) or insurer (4.0%). Several different types of patient experiences with trastuzumab biosimilars were reported. In Table 3, we characterize these scenarios identified in the dataset along with illustrative quotes.

**Table 3:** Types of experiences with trastuzumab biosimilars reported by patients

Patient experiences with trastuzumab biosimilars	Quotes
Unaware of trastuzumab biosimilars	*I haven't thought about until now. I thought kajinti was the same*
Started with trastuzumab biosimilar	*I have only ever been offered kanjinti. They talk to me about it as if it IS herceptin. It was never a choice offered (either way)*
Did not switch from reference to trastuzumab biosimilar	Not present in dataset
Switched from reference to trastuzumab biosimilar WITH notification	*My nurse informed me of the switch*
Declined the switch (or wanted to decline) to trastuzumab biosimilar	*If my insurance was willing to cover, my doctor should have respected my decision NOT to switch* *Chose not to switch*
Switched from reference to trastuzumab biosimilar WITHOUT notification	*Literally any information would have been more than I was given* *No one discussed w me*
Noticed on their own	*I always check my meds and asked why it was called something different* *I saw it on my chart* *I didn't know until I was reading insurance papers and the new drug was listed, so I emailed my dr and the pharmacist called and said she forgot to call me to tell me or something*
Switched back due to side-effects	*I was switched back to Herceptin after experiencing severe side-effects which I did not experience with Herceptin*
Switched between two biosimilars	Not present in dataset

Oncologists reported that the decision to switch a patient from biosimilar trastuzumab was not initiated by them (0.0%), but rather by the insurer (45.2%), pharmacy (29.0%), or hospital/center administration (19.4%). They reported that the most communication about the switch took place face-to-face (58.1%) and the 3 most common reasons for the switch included that the patient’s insurance requires a switch to a biosimilar (23.5%), it is the same treatment (14.1%), and that the substitution will save the hospital money (12.9%). Approximately 54.8% reported not receiving any pharmaceutical manufacturer material related to the selected biosimilar and 20.0% shared resources through a conversation with their patient.

### Patient-oncologist matching items

Using a scale of 0 (Disagree) to 100 (Agree), we administered 13 matching items to patients about their experience with trastuzumab biosimilars and oncologists about their perception of their patients’ experiences. Patients reported lower ratings (indicating less positive experience) (*M* = 43.74, *SD* = 18.94) than oncologists (*M* = 56.66, *SD* = 11.50). To calculate proportions, we recoded the data from continuous to categorical. Specifically, a score of > 50 was defined as positive and ≤ 50 was defined as negative (after accounting for items that needed to be reverse coded). For all but 1 item (patient understanding of switch), a smaller proportion of patients than oncologists responded positively. The top 5 items for which oncologists responded more positively than patients are the following: opportunity to ask questions, adequacy of resources, effectiveness of treatment, patient worry, and magnitude of change. See results in Table 4. Note, items in the table are slightly reworded from the original surveys to allow patient and oncologist results to be presented together.

**Table 4:** Patient-oncologist matching items

	Patients	Oncs	Diff (%)
(1) The switch to biosimilar trastuzumab was explained in an easily understood way	42.3% (63/149)	57.6% (19/33)	15.3
(2) The patient felt involved in this treatment decision to use biosimilar trastuzumab	18.4% (28/152)	18.2% (6/33)	− 0.2
(3) The oncologist is trusted to make the right decision about using biosimilar trastuzumab	62.6% (92/147)	66.7% (22/33)	4.1
(4) The insurance company is trusted to make the right decision about using biosimilar trastuzumab	9.3% (14/150)	12.1% (4/33)	2.8
(5) The hospital/center is trusted to make the right decision about using biosimilar trastuzumab	41.3% (59/143)	44.1% (15/34)	2.9
(6) The patient had the opportunity to ask questions about a switch to biosimilar trastuzumab	35.3% (54/153)	58.8% (20/34)	23.5
(7) The patient was given adequate resources about biosimilar trastuzumab to feel comfortable with this switch	17.6% (27/153)	33.3% (11/33)	15.7
(8) The cancer is/will be treated as effectively with biosimilar trastuzumab	43.4% (62/143)	79.4% (27/34)	36.1
(9) The patient understands the reason for this switch to biosimilar trastuzumab	42.6% (63/148)	39.4% (13/33)	-3.2
(10) The patient is worried about this switch to biosimilar trastuzumab (r)	23.6% (35/148)	41.2% (13/34)	17.5
(11) The patient has emotionally adjusted to this treatment switch that wasn’t due to cancer progression or quality of life issues	41.0% (59/144)	42.4% (14/33)	1.5
(12) The patient worries more about treatment success since this switch to biosimilar trastuzumab (r)	29.1% (43/148)	33.3% (11/33)	4.3
(13) Switching to a biosimilar trastuzumab is a minor change in the patient’s care	36.6% (53/145)	66.7% (22/33)	30.1
(r) reverse coded items			

### Patient self-reported information preference and seeking behaviors

About 61.0% of patients reported learning about biosimilars primarily through self-directed learning, 33.3% through a conversation with their healthcare provider, and 35.2% (56/159) through asking on social media. Approximately 55.6% reported wanting to: have time to discuss with their treating physician, achieve a better understanding of biosimilars (52.5%) and have access to printed materials (41.3%) and, specifically, ones that are user-friendly (23.1%).

## Discussion

This is the first set of surveys to evaluate communication gaps regarding trastuzumab biosimilars. Our findings reveal a lack of synchronicity between actual patient experiences and stated goals of oncology care, as evidenced in current practices of switching to biosimilars, at times, with lacking and/or inconsistent patient notification. We report three categories of findings: (1) types of patient experiences, (2) lack of common ground between patients and oncologists, and (3) patients and oncologists highlighting the need for more information.

### To inform tailored interventions, need to elicit and characterize patient experiences

Patient reported a variety of experiences with trastuzumab biosimilars, including being switched without notification, resulting in negative emotions*.* Some patients noticed on their own that they had been switched—in their chart or by reading insurance papers. This is evidence for a lack of consistent processes in how healthcare systems are managing communication with patients regarding switches to trastuzumab biosimilars, a systems-level issue. Furthermore, these experiences are inconsistent with best practices of patient-centered communication. A 2013 narrative review reported providing information and responding to emotions as two (among others) best practices in physician communication [18]. Switching to trastuzumab biosimilars for patients with breast cancer is a problem space in need of effective communication solutions.

### Patient-oncologist relationship

#### Oncologists need information

The objective of the oncologist survey was two-fold—not only to compare with patient responses, but to identify patient care barriers and gaps experienced by oncologists. Oncologist responses highlight that the decision to switch patients to trastuzumab biosimilars is often not made by them, but rather by the healthcare systems or by insurance companies. Some oncologists reported that this switch was done without their knowledge. Despite their central role in cancer patient care, oncologists lack complete information. It is important to identify what information oncologists need both for their own situational awareness and to foster relationships with their patients. Ultimately, effective communication about trastuzumab biosimilars is not the sole responsibility of individual oncologists, but rather an objective in need of systems-level interventions.

#### Patient and oncologists need to be on the same page

Patients and oncologists responded similarly (on the negative end of the scale) to items on the following topics: patient involvement, patient emotional adjustment, and hospital being trusted. These findings provide insight into operational gaps on which both patients and oncologists agree. In light of documented physician burnout [19] related to the pandemic as well as issues such as longer hours and higher volume of clerical tasks [20], the negative response of surveyed oncologists to trusting the hospital/center to make the right decision about using biosimilar trastuzumab is telling. Our work identifies an opportunity for better system support for both oncologists and patients. In the other two instances of agreement, the oncologist responses provide insight into appreciation that patients are impacted by the communication (or lack thereof) that underlies decision-making processes.

However, our data otherwise suggest that patients and oncologists are not on the same page. Specifically, the five topics that yielded different responses (with patients’ responses being more negative than oncologists’) are the following: (1) patient opportunity to ask questions; (2) patient receiving adequate resources; (3) cancer being treated as effectively; (4) patient being worried; and (5) the switch being a minor change. These findings highlight that there may be a fundamental lack of knowledge and comprehension by patients regarding the safety and efficacy of trastuzumab biosimilars, as well as a lack of appreciation by oncologists of the impact to the patient on switching medications. Patient educational materials may have the potential to put some patient concerns to rest, thereby alleviating the need for more time to ask questions. However, the patient responses regarding the importance of this switch to their care does not reveal *why* this change is perceived as important and further conversation may be needed between the oncologist and the patient.

#### Patients need information to mitigate patient work

Only 11% of surveyed patients reported being satisfied with the information they received. Patients in our data set report wanting to have information on trastuzumab biosimilars, such as printed materials (32%), more time between notification and treatment (35%), and better understanding of role of biosimilars in treatment (40%). Approximately 46% of patients reported learning more about biosimilars through self-directed learning on the internet than through oncologist/provider-directed methods. We also identified evidence of negative emotions regarding biosimilar switching. These findings suggest that ineffective communication about trastuzumab biosimilars contributes to patient work. Defined as behavioral, cognitive, and emotional effort exerted by the patient in navigating not just their care, but their life, patient work is a construct that has been receiving attention in the last decade [21]. Examples include information seeking, navigating associated relationships and logistics of doing so, and managing emotions—all activities that are time consuming and effortful [22].

Further, self-directed information seeking (outside of the healthcare team) suggests that the healthcare team is not central to the patient’s understanding of their care, thereby making the patient more vulnerable to misinformation, incomplete information, and lack of comprehension.

In addition, patients have the right to be informed regarding their treatment. Thus, the issue of lack of notification about switching to trastuzumab biosimilars is an ethical one. Lack of notification equates to lack of choice and opportunity to engage, as well as the absence of shared decision-making (or the perception of) between the patient and their care team. Without information about their care, patients do not have agency, further conflicting with the goals of patient-centered care.

#### Study limitations and strengths

Several study limitations need to be considered. Challenges in recruiting necessitated a lengthy timeline for data collection. We believe that recruitment challenges resulted from survey fatigue during the COVID-19 pandemic [23], as well as the nature of the research question that impacts a relatively small proportion of patients with breast cancer. Patient responses were limited to those who are active on social media resulting in a convenience sample. Further, the patient sample includes a low proportion of minorities, limiting the ability to generalize findings beyond White patients. However, although convenience samples have limited generalizability, they can be useful in understanding a phenomenon of interest. Specifically, recent research highlights that convenience samples correlate with random probability samples [24, 25]. Additionally, it is possible that the sample was biased in that people who have experienced challenges with trastuzumab biosimilars may have self-selected for the survey. Oncologist responses comprised a limited sample that are a reflection of the difficulty in recruiting healthcare providers for research studies [26, 27].

The strength of the current study is that it is the first of its kind to elicit and characterize patient and oncologist experiences in non-medical switching to trastuzumab biosimilars, and to compare those with oncologist perception of the patient experience.

#### Directions for future research

Research is needed to further investigate the role of trust in patient-oncologist relationships, and the impact of effective communication on patient emotional response. Literature suggests that trust, a dynamic and evolving process, is central to the patient-provider relationship [28]. Healthcare communication studies have highlighted the positive relationship between communication and trust [29]. In future research, it is important to explore the impact of the various patient experiences with trastuzumab biosimilars on the patient-oncologist relationship, perception of shared decision-making, being part of a team (characterized by common goals and mutual dependencies), and agency.

Critically, there is an urgent need to mitigate avoidable emotional distress to patients—especially populations that are already vulnerable on multiple levels. By characterizing the negative emotional consequences such as feelings of vulnerability and powerlessness of not being informed and engaged, we can begin to work toward tailored communication interventions. Not only is mental health at stake, but literature highlights its relationship with health behaviors including adherence to treatment [30].

Finally, to develop tailored interventions, it is necessary to characterize the experience of not just clinicians (as traditional) but patients and their families and caregivers through research methods such as surveys and interviews focused on eliciting lived experiences and unpacking decision points and knowledge gaps [31]. Findings should be used to inform the development and evaluation of interventions, resulting in practices that are tailored to the needs of patients and clinicians [32].

## Conclusion

We highlighted a discrepancy between patient-reported experiences and oncologist perceptions of the patient experience, suggesting that lack of adequate information is a challenge not only to the uptake of trastuzumab biosimilars, but to the patient-oncologist relationship. These findings not only have the potential to inform more effective communication regarding trastuzumab biosimilars but extend to communication regarding treatment options in general.

In conclusion, through tailored education and communication interventions, patients who currently have access to treatments will benefit by gaining awareness and understanding of trastuzumab biosimilars potentially contributing to maintenance of trust, engagement in shared decision-making and less likelihood to decline treatment with biosimilars, as well as improved healthcare communication [33].

## Supplementary Information

Below is the link to the electronic supplementary material.

Supplementary file1 (PDF 93 kb)

## Data Availability

The datasets generated during and/or analyzed during the current study are not publicly available but are available from the corresponding author on reasonable request.
