# Supplementary material for: Characterizing experiences of non-medical switching to trastuzumab biosimilars using data from internet-based surveys with US-based oncologists and breast cancer patients
Source: Breast Cancer Res Treat. 2022 May 14;194(1):25–33. doi: 10.1007/s10549-022-06615-2 (PMC9107314; doi:10.1007/s10549-022-06615-2)
Supplement: Supplementary file 1 — Supplementary file1 (PDF 93 kb) [file 10549_2022_6615_MOESM1_ESM.pdf]

Biosimilars (Patient)

Thank you for agreeing to participate in this research study to help us characterize communication, decision making roles, and processes associated with the switch from Herceptin (trastuzumab) to a biosimilar trastuzumab for treating HER2 positive breast cancer.

This study is being conducted by Dr. Elizabeth Papautsky at the University of Illinois at Chicago; Dr. Maryam Lustberg at Ohio State University; and patient advocate Martha Carlson. This study is funded by Pfizer. The study has been reviewed by the Institutional Review Board at the University of Illinois at Chicago and has been determined to be exempt (UIC Research Protocol #2020-0859; Exemption Granted Date: 7/29/2020).

To participate, you must be a breast cancer patient/survivor, have been diagnosed with HER2 positive breast cancer of any stage, be at least 18 years old, have either experienced a switch from Herceptin to a biosimilar or been presented with an option to switch, and be located in the US.

The questionnaire takes approximately 5-10 minutes to complete. You can skip any question you are uncomfortable answering. The data will be used for research purposes only. Results will be used to characterize treatment changes for individuals with HER2 positive breast cancer being treated with trastuzumab and/or biosimilar trastuzumab and develop interventions to facilitate such switches. Confidentiality will be maintained to the degree permitted by the technology used. Your participation in this online questionnaire involves risks similar to a person's everyday use of the Internet.

If you have any questions or comments about the study, please contact Dr. Elizabeth Papautsky at elp@uic.edu. If you have questions about your rights as a study subject; including questions, concerns, complaints, or if you feel you have not been treated according to the description in this form; or to offer input you may call the UIC Office for the Protection of Research Subjects (OPRS) at 312-996-1711 or 1-866-789-6215 (toll-free) or e-mail OPRS at uicirb@uic.edu.

For more information on HER2 positive breast cancer, trastuzumab, and biosimilar trastuzumab, please visit the US Food and Drug Administration and National Cancer Institute websites.

Please proceed to the questionnaire.

Dear Participant,

Treatment of HER2 positive breast cancer has recently seen the advent of biosimilars, including biosimilar trastuzumab products Kanjinti, Ontruzant, Ogivri, Trazimera, and Herzuma. A biosimilar is a biological medication. It is highly similar to a reference biological medication already approved by the FDA. For this study, this reference biological medication is Herceptin. Biosimilars are made from the same types of sources (e.g. living cells or microorganisms) as the reference medication but cannot be exactly copied.

We are interested in learning about your experience with the treatment of HER2 positive breast cancer. We appreciate your time and input. Please continue to the questionnaire.

Reminder: To participate, you must be a breast cancer patient/survivor, have been diagnosed with HER2 positive breast cancer of any stage, be at least 18 years old, have either experienced a switch from Herceptin to a biosimilar or been presented with an option to switch, and be located in the US.

Treatment

What is the current stage of your cancer?	◯ Stage I<br>◯ Stage II<br>◯ Stage III<br>◯ Stage IV/Metastatic<br>◯ Don't know
Are you currently receiving treatment or have received treatment in the last 1 year for HER2 positive breast cancer?	◯ Yes<br>◯ No<br>◯ Not Sure
Are you currently receiving or have received in the last 1 year trastuzumab (Herceptin) infusions?	◯ Yes<br>◯ No<br>◯ Not sure
Have you been presented with an option to switch your treatment from trastuzumab (Herceptin) to biosimilar trastuzumab?	◯ Yes<br>◯ No<br>◯ Not sure
Has your treatment switched from trastuzumab (Herceptin) infusion to a biosimilar trastuzumab?	◯ Yes<br>◯ No - I was presented with an option to switch to biosimilar trastuzumab, but chose to refuse<br>◯ No - I was not presented with an option to switch to biosimilar trastuzumab<br>◯ Not sure
What is the name of the biosimilar trastuzumab?	◯ Herzuma (trastuzumab-pkrb)<br>◯ Kanjinti (trastuzumab-anns)<br>◯ Ogivri (trastuzumab-dkst)<br>◯ Ontruzant (trastuzumab-dttb)<br>◯ Trazimera (trastuzumab-qyyp)<br>◯ I don't know

Demographics

What is your current age?

____

How many years has it been since your first diagnosis of HER2 positive breast cancer?

____

If relevant, how many years has it been since your diagnosis of HER2 positive stage IV/metastatic breast cancer?

____

Please specify your race. Please select as many as apply.

- ☐ American Indian or Alaskan Native
- ☐ Asian or Pacific Islander
- ☐ Black
- ☐ White
- ☐ Prefer not to answer

Please specify your ethnicity:

- ○ Hispanic origin
- ○ Not of Hispanic origin
- ○ Prefer not to answer

Please specify your gender:

- ○ Male
- ○ Female
- ○ Prefer not to answer
- ○ Other - please specify below

If other, please specify:

____

Where do you receive your HER2 positive breast cancer therapy infusions?

- ○ University-affiliated cancer center or its satellite location
- ○ Non-university affiliated cancer center (e.g. Cancer Centers of America, etc.)
- ○ Oncologist's office or community oncology setting
- ○ Community hospital
- ○ Veterans Affairs (VA) hospital
- ○ Not sure
- ○ Other - please specify below

If other, please specify:

____

What is the highest degree or level of education you have completed?

- ○ Some high school, no diploma
- ○ High school graduate, diploma or the equivalent (for example: GED)
- ○ Some college credit, no degree
- ○ Trade/technical/vocational training
- ○ Associate degree
- ○ Bachelor's degree
- ○ Master's degree
- ○ Professional degree
- ○ Doctorate degree

What is your current work situation?	◯ Retired<br>◯ Work part-time<br>◯ Work full-time<br>◯ Self-employed<br>◯ Unemployed<br>◯ Other or more detail - please specify below
If other, please specify:	______________________________
If applicable, what type of work do you do?	________________________

Please specify the US state or territory in which you reside?	◯ Alabama<br>◯ Alaska<br>◯ American Samoa<br>◯ Arizona<br>◯ Arkansas<br>◯ California<br>◯ Colorado<br>◯ Connecticut<br>◯ Delaware<br>◯ District of Columbia<br>◯ Florida<br>◯ Georgia<br>◯ Guam<br>◯ Hawaii<br>◯ Idaho<br>◯ Illinois<br>◯ Indiana<br>◯ Iowa<br>◯ Kansas<br>◯ Kentucky<br>◯ Louisiana<br>◯ Maine<br>◯ Maryland<br>◯ Massachusetts<br>◯ Michigan<br>◯ Minnesota<br>◯ Mississippi<br>◯ Missouri<br>◯ Montana<br>◯ Nebraska<br>◯ Nevada<br>◯ New Hampshire<br>◯ New Jersey<br>◯ New Mexico<br>◯ New York<br>◯ North Carolina<br>◯ North Dakota<br>◯ Northern Mariana Islands<br>◯ Ohio<br>◯ Oklahoma<br>◯ Oregon<br>◯ Pennsylvania<br>◯ Puerto Rico<br>◯ Rhode Island<br>◯ South Carolina<br>◯ South Dakota<br>◯ Tennessee<br>◯ Texas<br>◯ Utah<br>◯ Vermont<br>◯ Virgin Islands<br>◯ Virginia<br>◯ Washington<br>◯ West Virginia<br>◯ Wisconsin<br>◯ Wyoming
What type of area do you receive you treatment in?	◯ Urban<br>◯ Suburban<br>◯ Rural

What kind of insurance do you have for your cancer treatment?	◯ Fully covered by private insurance<br>◯ Partially covered by private insurance<br>◯ Medicare<br>◯ Medicaid<br>◯ Self-Pay<br>◯ Uninsured/No insurance<br>◯ Other - please specify below
If other, please specify:	__________________________________

Biosimilars

Question	Response
If your doctor has switched you from trastuzumab (Herceptin) to a biosimilar trastuzumab, how many months had you been receiving Herceptin and not a biosimilar?	______________________________
Who first discussed the treatment switch with you?	◯ Treating physician or oncologist<br>◯ Other physician<br>◯ Advanced practice providers (physician's assistant, nurse practitioner)<br>◯ Insurer<br>◯ Hospital<br>◯ Chemotherapy nurse<br>◯ Pharmacist<br>◯ I did not receive prior notification<br>◯ Other - please specify below
If other, please specify:	______________________________
In the past 12 months, the option to switch from Herceptin to biosimilar trastuzumab was most frequently initiated by:	◯ My oncologist<br>◯ Myself (patient)<br>◯ My (patient's) insurer<br>◯ Hospital/Center administration<br>◯ The pharmacy<br>◯ Other - please specify below
Other. Please specify:	______________________________
How did the communication about a treatment switch to a biosimilar trastuzumab take place?	◯ Video telehealth call<br>◯ Phone call<br>◯ Face-to-face<br>◯ Text<br>◯ Email<br>◯ Patient portal message<br>◯ Other - please specify below
If other, please specify:	______________________________
What reason(s) were you given for a treatment switch to a biosimilar trastuzumab? Please select as many as apply.	☐ It is the same treatment<br>☐ This substitution will save the hospital money because Herceptin is costly<br>☐ This substitution will save the cost of nursing staff as they spend time preparing you for an infusion<br>☐ This substitution will save me (the patient) money<br>☐ Other hospitals are switching to biosimilars<br>☐ Your insurance will no longer cover Herceptin and requires this treatment center to substitute a biosimilar<br>☐ Other - please specify below
If other, please specify:	______________________________

**If you have switched or have been provided with an option to switch to biosimilar trastuzumab, please rate (by sliding the bar anywhere along the range) your level of agreement on the following statements. Please note, you can use the ENTIRE RANGE OF THE SCALE.**	
My oncologist explained a switch to biosimilar trastuzumab in a way that was easy to understand	Strongly Disagree Neutral Strongly Agree<br>*(Place a mark on the scale above)*
I feel/felt involved in this treatment decision to choose a biosimilar trastuzumab for me	Strongly Disagree Neutral Strongly Agree<br>*(Place a mark on the scale above)*
I trust my oncologist in making the right decision to choose a biosimilar trastuzumab for me	Strongly Disagree Neutral Strongly Agree<br>*(Place a mark on the scale above)*
I trust my insurance company in making the right decision to require biosimilar trastuzumab instead of Herceptin	Strongly Disagree Neutral Strongly Agree<br>*(Place a mark on the scale above)*
I trust my hospital/center in making the right decision to require biosimilar trastuzumab instead of Herceptin	Strongly Disagree Neutral Strongly Agree<br>*(Place a mark on the scale above)*
I was given the opportunity to ask questions about a switch to biosimilar trastuzumab	Strongly Disagree Neutral Strongly Agree<br>*(Place a mark on the scale above)*
I was given adequate resources on biosimilar trastuzumab to feel comfortable with a switch	Strongly Disagree Neutral Strongly Agree<br>*(Place a mark on the scale above)*
My cancer is/will be treated as effectively with biosimilar trastuzumab as with Herceptin	Strongly Disagree Neutral Strongly Agree<br>*(Place a mark on the scale above)*
I understand the reason for switching to biosimilar trastuzumab	Strongly Disagree Neutral Strongly Agree<br>*(Place a mark on the scale above)*
This treatment switch to biosimilar trastuzumab makes me worried	Strongly Disagree Neutral Strongly Agree<br>*(Place a mark on the scale above)*

I have emotionally adjusted to this treatment switch that wasn't due to cancer progression or quality of life issues	Strongly Disagree Neutral Strongly Agree<br>*(Place a mark on the scale above)*
I worry more about my treatment success since this switch to a biosimilar trastuzumab	Strongly Disagree Neutral Strongly Agree<br>*(Place a mark on the scale above)*
Switching to a biosimilar trastuzumab is a minor change to my cancer care	Strongly Disagree Neutral Strongly Agree<br>*(Place a mark on the scale above)*

Information about biosimilars

Please indicate who most guided the decision associated with the switch to biosimilar trastuzumab<br><br>If your response is 'Not Applicable,' please leave the slider untouched and indicate 'NA' in the next question	Myself — Shared Decision — Healthcare System (provider, pharmacy, insurer, etc.)<br>*(Place a mark on the scale above)*
If your response to the previous item is 'Not Applicable,' please indicate as NA.	________________________________
I learned more about biosimilar trastuzumab through: Please select as many as apply.	☐ Conversation with my healthcare provider<br>☐ Resources given to me at a healthcare appointment<br>☐ Resources at my treatment center<br>☐ Provider-directed research on the internet<br>☐ Self-directed research on the internet<br>☐ Asking on social media (Facebook, Twitter, etc) patient communities and cancer sites<br>☐ Other/additional information - please specify below
Please share any additional information:	________________________________
What would have helped your understanding of this treatment switch to biosimilar trastuzumab? Please select as many as apply.	☐ None, I'm satisfied with the information I received through my provider<br>☐ More time to discuss the biosimilar trastuzumab with my treating physician<br>☐ More printed material about the biosimilar trastuzumab<br>☐ More user-friendly material about biosimilar trastuzumab<br>☐ More time between notification of the change and the initial treatment with the biosimilar trastuzumab<br>☐ More understanding about the role of biosimilars in treatment of HER2 positive breast cancer<br>☐ Other/additional information - please specify below
Please share any additional information:	________________________________
Thinking about this treatment switch to biosimilar trastuzumab, what considerations aided your decision? Please select as many as apply.	☐ Better quality of life<br>☐ Lower drug/treatment cost<br>☐ Shorter infusion time<br>☐ Encouragement from my caregiver(s) (partner, etc)<br>☐ I did not have a choice<br>☐ Other/additional information - please specify below
Please share any additional information:	________________________________
Please expand on aspects of your treatment switch to a biosimilar trastuzumab that you consider especially important:	____

Thank you very much for your time and input!

Biosimilars (Physician)

Thank you for agreeing to participate in this research study to help us characterize communication, decision making roles, and processes associated with the transition from Herceptin (trastuzumab) to a biosimilar for treating HER2 positive breast cancer care.

This study is being conducted by Dr. Elizabeth Papautsky at the University of Illinois at Chicago; Dr. Maryam Lustberg at Ohio State University; and patient advocate Martha Carlson. This study is funded by Pfizer. The study has been reviewed by the Institutional Review Board at the University of Illinois at Chicago and has been determined to be exempt (UIC Research Protocol #2020-0859; Exemption Granted Date: 7/29/2020).

To be eligible to participate, you must be a US-based physician who has prescribed medical treatment for persons diagnosed with HER2 positive breast cancer, any stage, within the past year.

The questionnaire takes approximately 5-10 minutes to complete. You can skip any question you are uncomfortable answering. The data will be used for research purposes only. Results will be used to characterize processes around treatment changes for individuals with HER2 positive breast cancer being treated with trastuzumab and/or biosimilar trastuzumab and develop interventions to facilitate such switches.

Confidentiality will be maintained to the degree permitted by the technology used. Your participation in this online questionnaire involves risks similar to a person's everyday use of the Internet.

If you have any questions or comments about the study, please contact Dr. Elizabeth Papautsky at elp@uic.edu. If you have questions about your rights as a study subject; including questions, concerns, complaints, or if you feel you have not been treated according to the description in this form; or to offer input you may call the UIC Office for the Protection of Research Subjects (OPRS) at 312-996-1711 or 1-866-789-6215 (toll-free) or e-mail OPRS at uicirb@uic.edu.

Please proceed to the questionnaire.

Dear Participant,

Treatment of HER2 positive breast cancer has recently seen the advent of biosimilars, including biosimilar trastuzumab products Kanjinti, Ontruzant, Ogivri, Trazimera, and Herzuma. A biosimilar is a biological medication. It is highly similar to a reference biological medication already approved by the FDA. For this study, this reference biological medication is Herceptin. Biosimilars are made from the same types of sources (e.g. living cells or microorganisms) as the reference medication but cannot be exactly copied.

We are interested in learning about your experience with the treatment of HER2 positive breast cancer. We appreciate your time and input. Please continue to the questionnaire.

Reminder: To be eligible to participate, you must be a US-based physician who has prescribed medical treatment for persons diagnosed with HER2 positive breast cancer, any stage, within the past year.

Are you a US-based physician?	◯ Yes<br>◯ No
Are you currently providing treatment for patients with HER2 positive breast cancer?	◯ Yes<br>◯ No<br>◯ Not sure

Treatment

Are you currently prescribing trastuzumab (Herceptin) infusions?	◯ Yes<br>◯ No<br>◯ Not sure
Have you switched your patients' breast cancer treatments from Herceptin infusion to a biosimilar trastuzumab?	◯ Yes<br>◯ No<br>◯ Not sure
What is/are the biosimilar trastuzumab(s) to which you've switched patients? Please select as many as apply.	☐ Herzuma (trastuzumab-pkrb)<br>☐ Kanjinti (trastuzumab-anns)<br>☐ Ogivri (trastuzumab-dkst)<br>☐ Ontruzant (trastuzumab-dttb)<br>☐ Trazimera (trastuzumab-qyyp)<br>☐ I don't know

Demographics

Number of years providing treatment for people with HER2 positive breast cancer:	______________ (years)
Please specify your race. Please select as many as apply.	☐ American Indian or Alaskan Native<br>☐ Asian or Pacific Islander<br>☐ Black<br>☐ White<br>☐ Prefer not to answer
Please specify your ethnicity:	◯ Hispanic origin<br>◯ Not of Hispanic origin<br>◯ Prefer not to answer
Please specify your gender:	◯ Male<br>◯ Female<br>◯ Prefer not to answer<br>◯ Other - please specify below
Other. Please specify:	______________
What is your current age?	______________ (years)
Where do most of your patients receive HER2 positive breast cancer therapy infusions? Please select as many as apply.	☐ University-affiliated cancer center or its satellite location<br>☐ Non-university affiliated cancer center (e.g. Cancer Centers of America, etc.)<br>☐ Oncologist's office or community oncology setting<br>☐ Community hospital<br>☐ Veterans Affairs (VA) hospital<br>☐ Not sure<br>☐ Other - please specify below
Other. Please specify:	______________
Where do you practice? Please select as many as apply.	☐ University-affiliated cancer center or its satellite location<br>☐ Non-university affiliated cancer center (e.g. Cancer Centers of America, etc.)<br>☐ Oncologist's office or community oncology setting<br>☐ Community hospital<br>☐ Veterans Affairs (VA) hospital<br>☐ Not sure<br>☐ Other - please specify below
Other. Please specify:	______________

Please specify the US state or territory in which you reside:	◯ Alabama<br>◯ Alaska<br>◯ American Samoa<br>◯ Arizona<br>◯ Arkansas<br>◯ California<br>◯ Colorado<br>◯ Connecticut<br>◯ Delaware<br>◯ District of Columbia<br>◯ Florida<br>◯ Georgia<br>◯ Guam<br>◯ Hawaii<br>◯ Idaho<br>◯ Illinois<br>◯ Indiana<br>◯ Iowa<br>◯ Kansas<br>◯ Kentucky<br>◯ Louisiana<br>◯ Maine<br>◯ Maryland<br>◯ Massachusetts<br>◯ Michigan<br>◯ Minnesota<br>◯ Mississippi<br>◯ Missouri<br>◯ Montana<br>◯ Nebraska<br>◯ Nevada<br>◯ New Hampshire<br>◯ New Jersey<br>◯ New Mexico<br>◯ New York<br>◯ North Carolina<br>◯ North Dakota<br>◯ Northern Mariana Islands<br>◯ Ohio<br>◯ Oklahoma<br>◯ Oregon<br>◯ Pennsylvania<br>◯ Puerto Rico<br>◯ Rhode Island<br>◯ South Carolina<br>◯ South Dakota<br>◯ Tennessee<br>◯ Texas<br>◯ Utah<br>◯ Vermont<br>◯ Virgin Islands<br>◯ Virginia<br>◯ Washington<br>◯ West Virginia<br>◯ Wisconsin<br>◯ Wyoming
What type of area do you practice in?	◯ Urban<br>◯ Suburban<br>◯ Rural

Over the past 12 months, how have your patients with HER2 positive breast cancer most often paid for their treatment expenses?	◯ Fully covered by private insurance<br>◯ Partially covered by private insurance<br>◯ Medicare<br>◯ Medicaid<br>◯ Self-Pay<br>◯ Uninsured/No insurance<br>◯ Not sure<br>◯ Other - please specify below
Other. Please specify:	______________________________

If you have switched patients with HER2 positive breast cancer from Herceptin to a biosimilar trastuzumab, please rate (by sliding the bar anywhere along the range) your level of agreement on the following statements. Please note, you can use the ENTIRE RANGE OF THE SCALE.

The decision to use a biosimilar trastuzumab was under my control

Strongly Disagree Neutral Strongly Agree

(Place a mark on the scale above)

I explained the switch to biosimilar trastuzumab in a way that was easy to understand

Strongly Disagree Neutral Strongly Agree

(Place a mark on the scale above)

I make/made sure that my patients feel/felt involved in this treatment decision to switch to biosimilar trastuzumab for them

Strongly Disagree Neutral Strongly Agree

(Place a mark on the scale above)

My patients trust me in making the right decision to choose a biosimilar trastuzumab for them

Strongly Disagree Neutral Strongly Agree

(Place a mark on the scale above)

My patients trust their insurance company in making the right decision to require biosimilar trastuzumab instead of Herceptin

Strongly Disagree Neutral Strongly Agree

(Place a mark on the scale above)

My patients trust their hospital/center in making the right decision to require biosimilar trastuzumab instead of Herceptin

Strongly Disagree Neutral Strongly Agree

(Place a mark on the scale above)

I give my patients an opportunity to ask questions about biosimilar trastuzumab

Strongly Disagree Neutral Strongly Agree

(Place a mark on the scale above)

I give my patients adequate resources on biosimilar trastuzumab to help with decision making

Strongly Disagree Neutral Strongly Agree

(Place a mark on the scale above)

Cancer is/will be treated as effectively with biosimilar trastuzumab as with Herceptin

Strongly Disagree Neutral Strongly Agree

(Place a mark on the scale above)

My patients understand the reason for switching to biosimilar trastuzumab

Strongly Disagree Neutral Strongly Agree

(Place a mark on the scale above)

This treatment switch to a biosimilar trastuzumab makes my patients feel worried	Strongly Disagree Neutral Strongly Agree<br>*(Place a mark on the scale above)*
My patients have emotionally adjusted to this treatment switch that wasn't due to cancer progression or quality of life issues	Strongly Disagree Neutral Strongly Agree<br>*(Place a mark on the scale above)*
My patients worry more about treatment success since this switch to a biosimilar trastuzumab	Strongly Disagree Neutral Strongly Agree<br>*(Place a mark on the scale above)*
Switching to a biosimilar trastuzumab is a minor change to cancer care of my patients	Strongly Disagree Neutral Strongly Agree<br>*(Place a mark on the scale above)*

Information about biosimilars

Please indicate who most guided the decision associated with the switch to biosimilar trastuzumab.<br><br>If your response is 'Not Applicable,' please leave the slider untouched and indicate 'NA' in the next question.	Patient — Shared Decision — Healthcare System (provider, pharmacy, insurer, etc.)<br><br>*(Place a mark on the scale above)*
If your response to the previous item is 'Not Applicable,' please indicate as NA.	______________________
In the past 12 months, the decision to switch a patient from Herceptin to biosimilar trastuzumab was most frequently initiated by:	◯ Myself<br>◯ The patient<br>◯ The patient's insurer<br>◯ Hospital/Center administration<br>◯ The pharmacy<br>◯ Other - please specify below
Other. Please specify:	______________________
How did most communications about this treatment switch to a biosimilar trastuzumab take place?	◯ Video telehealth call<br>◯ Phone Call<br>◯ Face to Face<br>◯ Text<br>◯ Email<br>◯ Patient portal message<br>◯ Other - please specify below
Other. Please specify:	______________________
What reason(s) informed this treatment switch to biosimilar trastuzumab?<br>Please select as many as apply.	☐ It is the same treatment<br>☐ This substitution will save the hospital money because Herceptin is costly<br>☐ This substitution will save the cost of nursing staff as they spend time preparing you for an infusion<br>☐ This substitution will save the patient money<br>☐ Other hospitals are switching to biosimilars<br>☐ The patient's insurance will no longer cover Herceptin and requires this treatment center to substitute a biosimilar<br>☐ Other - please specify below
Other. Please specify:	______________________
Did you receive pharmaceutical manufacturer material related to the selected biosimilar trastuzumab?	◯ Yes<br>◯ No<br>◯ Not sure
I shared the resources with my patients about this biosimilar trastuzumab through the following. Please select as many as apply.	☐ Conversation with my patient<br>☐ Resources provided by pharmaceutical company<br>☐ Resources created/provided by my practice<br>☐ Directing my patient to appropriate online sites<br>☐ Other - please specify below

Other. Please specify:	________________________________
Please expand on aspects of treatment decisions related to biosimilar trastuzumab that you consider especially important:	____

Thank you very much for your time and input!
